# The Influence of Distance and Level of Care on Delivery Place in Rural Zambia: A Study of Linked National Data in a Geographic Information System

**DOI:** 10.1371/journal.pmed.1000394

**Published:** 2011-01-25

**Authors:** Sabine Gabrysch, Simon Cousens, Jonathan Cox, Oona M. R. Campbell

**Affiliations:** 1 Institute of Public Health, Ruprecht-Karls-Universität, Heidelberg, Germany; 2 Faculty of Epidemiology and Population Health, London School of Hygiene & Tropical Medicine, London, United Kingdom; 3 Faculty of Infectious and Tropical Diseases, London School of Hygiene & Tropical Medicine, London, United Kingdom; Johns Hopkins Bloomberg School of Public Health, United States of America

## Abstract

**Background:**

Maternal and perinatal mortality could be reduced if all women delivered in settings where skilled attendants could provide emergency obstetric care (EmOC) if complications arise. Research on determinants of skilled attendance at delivery has focussed on household and individual factors, neglecting the influence of the health service environment, in part due to a lack of suitable data. The aim of this study was to quantify the effects of distance to care and level of care on women's use of health facilities for delivery in rural Zambia, and to compare their population impact to that of other important determinants.

**Methods and Findings:**

Using a geographic information system (GIS), we linked national household data from the Zambian Demographic and Health Survey 2007 with national facility data from the Zambian Health Facility Census 2005 and calculated straight-line distances. Health facilities were classified by whether they provided comprehensive EmOC (CEmOC), basic EmOC (BEmOC), or limited or substandard services. Multivariable multilevel logistic regression analyses were performed to investigate the influence of distance to care and level of care on place of delivery (facility or home) for 3,682 rural births, controlling for a wide range of confounders. Only a third of rural Zambian births occurred at a health facility, and half of all births were to mothers living more than 25 km from a facility of BEmOC standard or better. As distance to the closest health facility doubled, the odds of facility delivery decreased by 29% (95% CI, 14%–40%). Independently, each step increase in level of care led to 26% higher odds of facility delivery (95% CI, 7%–48%). The population impact of poor geographic access to EmOC was at least of similar magnitude as that of low maternal education, household poverty, or lack of female autonomy.

**Conclusions:**

Lack of geographic access to emergency obstetric care is a key factor explaining why most rural deliveries in Zambia still occur at home without skilled care. Addressing geographic and quality barriers is crucial to increase service use and to lower maternal and perinatal mortality. Linking datasets using GIS has great potential for future research and can help overcome the neglect of health system factors in research and policy.

*Please see later in the article for the Editors' Summary*

## Abstract 2

Using linked national data in a geographic information system system, Sabine Gabrysch and colleagues investigate the effects of distance to care and level of care on women's use of health facilities for delivery in rural Zambia.

## Editors' Summary

**Background:**

Approximately 360,000 women die each year in pregnancy and childbirth, of which more than 200,000 in sub-Saharan Africa, where a woman's lifetime risk of dying during or following pregnancy remains as high as 1 in 31 (compared to 1 in 4,300 in the developed world). The target of Millennium Development Goal 5 is to reduce the maternal mortality ratio by three quarters by 2015. Most maternal and neonatal deaths in low-income countries could be prevented if all women delivered their babies in settings where skilled birth attendants (such as midwives) were available and could provide emergency obstetric care to both mothers and babies in case of complications. Yet every year roughly 50 million women give birth at home without skilled care.

**Why was this study done?:**

The likelihood of a woman giving birth in a health facility under the care of a skilled birth attendant depends on many factors. These include characteristics of the mother and her family, such as education level and household wealth, and aspects of the health service environment—distance to the nearest health facility and the quality of care provided at that facility, for example. However, research to date has typically focused on household and individual factors, neglecting the influence of the health service environment on choice of delivery place, largely because suitable data was not available. In this study in rural Zambia, the researchers aimed to quantify the effects of the health service environment, namely distance to health care and the level of care provided, on pregnant women's use of health facilities for giving birth. To put these factors in context, the researchers compared the impact of distance to quality care on place of delivery to that of other important factors, such as poverty and education.

**What did the researchers do and find?:**

Using a geographic information system (GIS), the researchers linked national household data (from the 2007 Zambia Demographic and Health Survey) with national facility data (from the 2005 Zambian Health Facility Census) and calculated straight-line distances between women's villages and health facilities. Health facilities were classified as providing comprehensive emergency obstetric care, basic emergency obstetric care, or limited or substandard services by using reported capability to perform a certain number of the eight emergency obstetric care signal functions: injectable antibiotics, injectable oxytocics, injectable anticonvulsants, manual removal of placenta, manual removal of retained products, assisted vaginal delivery, cesarean section, and blood transfusion, as well as criteria on staffing, opening hours and referral capacity. The researchers used data from 3,682 rural births and multivariable multilevel logistic regression analyses to investigate whether distance to, and level of care at the closest delivery facility influence place of delivery (health facility or home), keeping other influential factors constant.

The researchers found that only a third of births in rural Zambia occurred at a health facility, and half of all mothers who gave birth lived more than 25 km from a health facility that provided basic emergency obstetric services. As distance to the closest health facility doubled, the odds of a women giving birth in a health facility decreased by 29%. Independently, each step increase in the level of emergency obstetric care provided at the closest delivery facility led to an increased likelihood (26% higher odds) of a woman delivering her baby at a facility. The researchers estimated that the impact of poor geographic access to emergency obstetric services was of similar magnitude as that of low maternal education, household poverty, or lack of female autonomy.

**What do these findings mean?:**

The results of this study suggest that poor geographic access to emergency obstetric care is a key factor in explaining why most women in rural Zambia still deliver their babies at home without skilled care. Therefore, in order to increase the number of women delivering in health facilities and thus reduce maternal and neonatal mortality, it is crucial to address the geographic and quality barriers to delivery service use. Furthermore, the methodology used in this study—linking datasets using GIS— has great potential for future research as it can help explore the influence of health system factors also for other health problems.

**Additional Information:**

Please access these websites via the online version of this summary at http://dx.doi.org/10.1371/journal.pmed.1000394.

- Information about emergency obstetric care is provided by the United Nations Population Fund (UNFPA)
- Various topics on maternal health are presented by WHO, WHO Regional Office Africa, by UNPFA, and UNICEF
- WHO offers detailed information about MDG5
- Family Care International offers more information about maternal and neonatal health
- The Averting Maternal Death and Disability program (AMDD) provides information on needs assessments of emergency obstetric and newborn care
- Countdown to 2015 tracks progress in maternal, newborn, and child survival
- WHO provides free online viewing of BBC Fight for Life videos describing delivery experiences in different countries

## Introduction

Maternal and perinatal mortality rates are still alarmingly high, especially in sub-Saharan Africa, where little progress has been made over recent decades [1]. Globally, an estimated 225,000 maternal deaths, 904,000 neonatal deaths, and 1.02 million stillbirths annually are intrapartum related [2]. Most of these deaths occur in low-income countries and could be avoided if all women delivered in a setting where skilled attendants can provide emergency obstetric care (EmOC) and life-saving neonatal interventions in the event of complications [2]–[4]. Yet every year 50 million women give birth at home without skilled care [5].

The factors influencing use of skilled attendants at delivery include demographic, socioeconomic, and other characteristics of the mother and her family, as well as aspects of the service environment such as distance to the nearest health facility and quality of care [6],[7]. While many epidemiological studies have investigated individual and household factors [7], “the context within which utilization occurs—the role of the environment and provider-related factors—has been largely neglected” [8], because studies collecting data on individual service use rarely also collect data on the health services available to these individuals [8],[9]. Geographical studies, on the other hand, generally evaluate accessibility factors without controlling for individual-level variables [10],[11]. Ignoring important determinants gives an incomplete picture and can lead to erroneous conclusions through uncontrolled confounding, which in turn may lead to setting the wrong priorities in public health policy.

The relatively small number of epidemiological studies on determinants of use of delivery services that consider the health service environment generally use two different approaches. One is to gather household and facility data from a small area, e.g., from a surveillance site. While this can provide detailed information, findings may be very specific and not easily generalisable. Furthermore, risk factors may not emerge as important due to a lack of variation in that setting, e.g., if geographic access is good throughout the area. The restricted number of facilities in small study areas also limits the suitability of this approach for studying the effect of level of care (i.e., staffing and types of services provided) on facility use. The other approach uses large-scale household surveys that collect additional information on the health facilities in the surroundings, e.g., from key informants in the Demographic and Health Survey (DHS) service availability module [12]. The information gathered on the facilities in this case is usually very limited—distance to the closest facility may be recorded but it is usually unclear what services are offered there. This leads to misclassification of distance to delivery care, and usually precludes study of the effect of level of care on facility use.

This trade-off between data scale and detail explains why there are “surprisingly few studies examining the effect of the level of functioning of health centres on utilisation of maternity care” [13], despite consistent qualitative evidence on the importance of quality of care for choice of delivery place, and why the effect of quality of care has not been clearly quantified to date.

A solution to this problem is to merge databases that include detailed individual-level data with databases that include data on services [8]. With the increasing availability of georeferenced data this is becoming a feasible option: if both large-scale household data and detailed health facility data include geographic coordinates, they can be linked in a geographic information system (GIS).

Many recent DHS household surveys have recorded the geographic coordinates of their sampled clusters, but detailed data on health facilities, including geographic information, are harder to come by. Zambia is one of few low-income countries for which both suitable facility and household data are available.

Like most sub-Saharan African countries, Zambia has a very high maternal mortality ratio, estimated to be 591 maternal deaths for every 100,000 live births in the 2007 DHS [14]. Around 65% of Zambia's 13 million people live in rural areas [15]. Half of all births in Zambia occur with a skilled attendant in a health facility – over 80% of births in urban areas but only about 30% in rural areas [14]. Unattended home deliveries are therefore largely a rural problem in Zambia.

The aim of this study was to quantify the influence of the health service environment on women's use of health facilities for delivery in rural Zambia, adjusting for other important individual-, household-, and community-level determinants.

The specific objectives of this work were to:

1. Estimate the effect of distance to the closest health facility offering delivery care on place of delivery,
2. Estimate the effect of the level of care offered at the closest health facility on place of delivery, and
3. Estimate the impact (population attributable fraction [PAF]) of distance to facilities capable of providing EmOC on place of delivery, in comparison to other important determinants of delivery service use.

## Methods

Ethical approval of this study was granted by the London School of Hygiene & Tropical Medicine ethics committee on 03 July 2007 (application number 5172).

The Zambia DHS 2007 contains information on 4,146 rural births (counting twins and triplets as one birth as they represent one delivery episode) that occurred in the five years before the survey (2002–2007). Of these, 454 births (11%) occurred prior to the mothers' moving to the current place of residence, and thus the distance and other cluster characteristics at time of interview were not those at the time of birth. Excluding these births left a sample of 3,692 births in 203 sampling clusters. For 3,682 of these, information on place of delivery (home or facility) was available. Detailed information about the Zambia DHS 2007 is available in the report [14] and at http://www.measuredhs.com.

The Zambian Health Facility Census (HFC) [16] 2005 covered all public and semipublic (e.g., mission or nongovernmental organization) facilities in the country as well as some larger private for-profit facilities. Functionality and level of EmOC were assessed using reported capability for eight EmOC signal functions: (1) injectable antibiotics, (2) injectable oxytocics, (3) injectable anticonvulsants, (4) manual removal of placenta, (5) manual removal of retained products, (6) assisted vaginal delivery, (7) cesarean section, and (8) blood transfusion. Ideally, actual performance of these signal functions in the previous three months should be used as indicators [17], but this was not ascertained in the Zambian HFC. It is known that reported theoretical capability overestimates actual functioning [18],[19]. Therefore, we added criteria on opening hours, staffing, electricity availability, and referral capacity to our EmOC classification.

Two main levels of care were defined, corresponding typically to hospitals and health centres: comprehensive EmOC (CEmOC) services imply provision of all eight signal functions and basic EmOC (BEmOC) services provision of the first six [19]. We additionally allowed for the signal function of assisted vaginal delivery, using either forceps or vacuum extractor, to be missing, as it would be misleading to discount facilities as EmOC just because they lack this one signal function that is not always routinely taught and performed [20]. These facilities are called CEmOC minus one (CEmOC−1) and BEmOC minus one (BEmOC−1) [20],[21]. Two further levels of care were defined for facilities not providing EmOC but nevertheless some useful services, termed BEmOC−2 and BEmOC−4 (lacking any two or four basic signal functions) [21].

Table 1 presents our criteria for determining the EmOC levels of the 90 hospitals, 990 health centres, and 50 health posts nationwide recorded as offering delivery care in the HFC dataset, and the number of facilities fulfilling these. Of 1,131 delivery facilities, 135 (12%) fulfilled CEmOC(−1) (i.e., either CEmOC or CEmOC−1) or BEmOC(−1) (BEmOC or BEmOC−1) criteria, while 466 (41%) did not fulfil even BEmOC−4 criteria and were thus classified as substandard.

**Table 1:** EmOC classification of Zambian health facilities.

Variable	CEmOC(−1)	BEmOC(−1)	BEmOC−2	BEmOC−4
**Signal functions** a	All eight, or all except assisted vaginal delivery (+ electricity)	All six basic, or all except assisted vaginal delivery	At least four basic functions	At least two basic functions
**Service hours**	Midwife/doctor present or on call 24 hours	Midwife/doctor present or on call 24 hours	Midwife/doctor present or on call 24 hours	Any health professional with midwifery skills present or on call 24 hours
**Staffing** b	≥2 doctors registered, ≥1 doctor on duty	≥3 health professionals registered, ≥1 health professional on duty	≥2 health professionals registered, ≥1 health professional on duty	≥1 health professional on duty
**Referral capacity**	Not required	Offer referral^c^, provide vehicle^d^, or have communication tool	Offer referral^c^, provide vehicle^d^, or have communication tool	Offer referral^c^, provide vehicle^d^, or have communication tool
**Facilities qualifying** e	54	81	155	375

a Six basic signal functions: Injectable antibiotics, injectable anticonvulsants, injectable oxytocics, manual removal of placenta, manual removal of retained products, assisted vaginal delivery. Two comprehensive signal functions: C-section, blood transfusion.

b Health professional: doctor, nurse, midwife or clinical officer. Registered: recorded as working in the facility. On duty: present at day of visit.

c Not required if offering comprehensive signal functions themselves.

d Not required if next door to a CEmOC(−1) facility.

e There was a total of 1,131 facilities offering delivery care. The remaining 466 facilities did not fulfil even BEmOC−4 criteria and were classified as substandard.

Straight-line distances in metres from each DHS cluster to the closest delivery facility of a certain level of care were calculated in the GIS platform ArcView using the extension “Nearest Neighbor 3.6,” and exported into the statistical software package Stata 10.1 for further analysis. It should be noted that DHS cluster coordinates contain up to 5 km of random error due to a “geo-scrambling procedure” employed by Macro International to ensure confidentiality [22]. The error introduced by this scrambling, together with a lack of data on roads and terrain, precluded a more accurate estimation of travel time.

**Figure 1:**
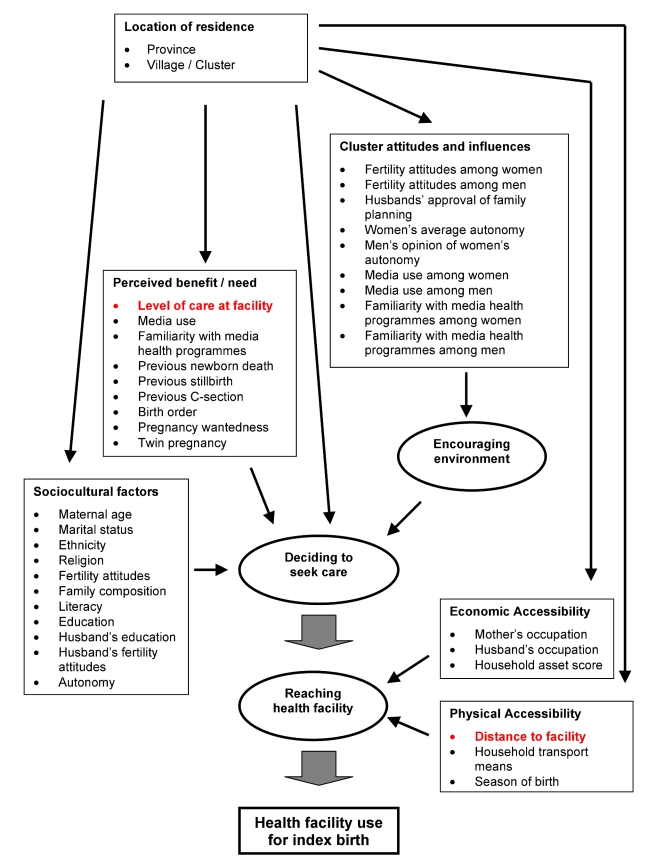
Conceptual framework of influences on health service use. According to the expanded “three delays model” [6],[7], the first delay is in making the decision to seek care and the second is in reaching the health facility, while the third is in receiving adequate care in the facility (not considered here). Sociocultural factors and perceived benefit and need of facility use influence the decision to seek care. Economic and physical accessibility mainly influence whether the woman actually reaches the facility (and perceived accessibility also influences decision-making). Furthermore, this framework considers how cluster or community attitudes create a more or less encouraging environment for family decision-making. The location of residence influences most other factors. The factors of interest to this study are highlighted in red.

The conceptual framework we used to guide our analysis is presented in Figure 1. The outcome of interest is facility delivery; the exposures of primary interest are distance to the closest delivery facility and level of EmOC available at the delivery facility. Nearly all variables are associated with place of residence and thus are potential confounders of the relationships of interest. Season of birth and household ownership of a means of transport were considered potential effect modifiers of distance.

To estimate households' ability to pay for the costs associated with facility delivery, we constructed a wealth index using 18 household assets, including farming assets, but excluding electricity and household means of transport, as these also capture aspects of infrastructure and geographic access. For each asset, the most expensive option (e.g., flush toilet) was given 10 points, the least expensive 0 points (e.g., no toilet facility), and other options (e.g., pit latrine) intermediate values. We used this simple weighting approach instead of principal component analysis (PCA), as it is more transparent and does not perform worse [23], and because PCA is problematic for discrete data [24]. Women's autonomy variables were constructed using information on age at marriage; decision-making power for purchases, visits, and health care; and women's opinion on the justification of wife-beating and justification of refusal of sex. Community-level variables were constructed using averages of all interviewed women in the cluster (not just those with births in the last five years) and of all interviewed men.

The dataset is hierarchically structured: A mother can have several births over five years, several mothers may belong to the same household, and many households make up a sampling cluster. We chose a three-level random-effects logistic regression model to account for the dependency between births to the same mother and in the same cluster in terms of facility delivery. We omitted the household level, as its influence was small and there was no evidence that it improved the model. The model was implemented using the “xtmelogit” command in Stata 10.1.

The association between distance and facility use was steeper for shorter distances and levelling off with larger distances, as might be expected. After a logarithmic transformation, the association was approximately linear, as assessed by lowess plots (see Figure S1). Level of care was included in the regression model as an indicator variable denoting the level of care available at the closest facility or within 10 km thereof, to account for the presence of a second higher-level facility at similar distance.

We first examined the influence of each potential confounder on the estimates for the exposures of primary interest. Variables changing the log(odds ratio) of distance or level of care by 10% or more were considered for inclusion in a multivariable model. This was built including education and household wealth as a priori confounders and then adding other variables in the order of the strength of their individual confounding effects. Variables were retained in the model if their inclusion altered by at least 10% one or both of the log(odds ratios) for the primary exposures. First, a model was developed that did not include cluster-level confounders and then a second model was developed including these. All *p-*values are two-tailed.

To estimate the PAFs, a full explanatory model was built containing all variables that independently influence facility use for delivery, to control for mutual confounding (instead of only including variables that confound the associations of distance and level of care with facility delivery). Distance was in the model a priori and variables were added in the order of their effect sizes and significance in univariable analysis, keeping and eliminating variables according to a cut-off significance level of *p* = 0.05 in Wald tests. PAFs and 95% confidence intervals were calculated with the user-written Stata command “aflogit,” which adjusts each PAF for all other variables in the model. This command does not run after multilevel models and therefore we used robust standard errors in this model instead.

## Results

Of the 3,692 births to rural mothers in the DHS 2007 with relevant distance information, 32.5% occurred in a health facility, 0.4% were home deliveries attended by a nurse or midwife, and 67.1% were neither in a facility nor attended professionally.

**Figure 2:**
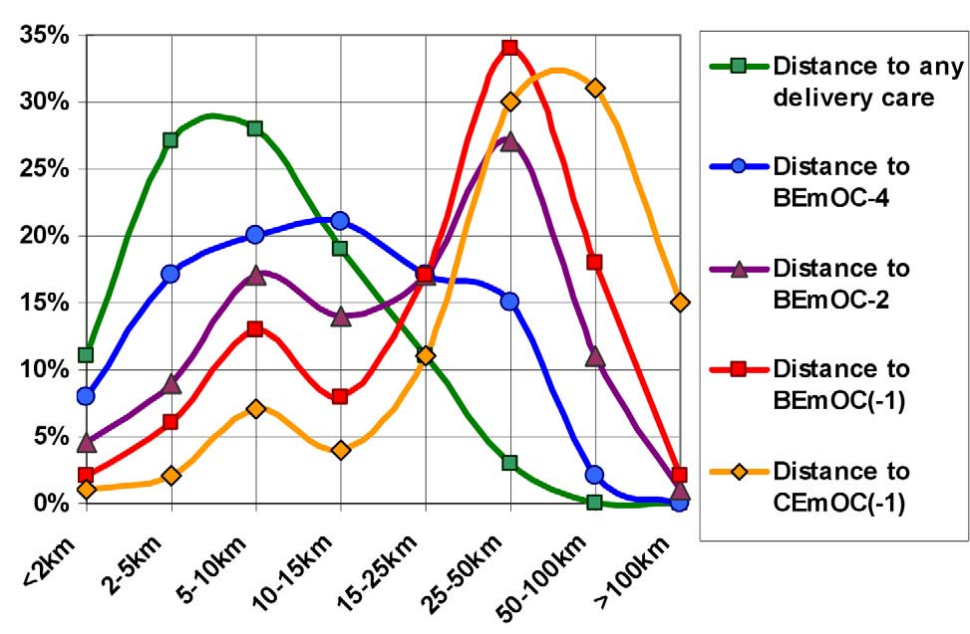
Distance distributions to different levels of delivery care in rural Zambia. Distance distributions to different levels of care are shown for 3,692 rural Zambian births from the 2007 DHS. While most births were within walking distance of a facility offering delivery care (green), distances to facilities offering EmOC functions were larger. Few births were within walking distance to basic (red) or comprehensive (yellow) EmOC, most being 25 km or more from such care. BEmOC(−1), basic emergency obstetric care that may lack assisted vaginal delivery; BEmOC−2 and −4, facilities lacking two or four of the BEmOC signal functions, respectively; CEmOC(−1), comprehensive emergency obstetric care that may lack assisted vaginal delivery. For details on the classification, see Methods.

Figure 2 shows how far the births were from different levels of care. While most births were within walking distance of a facility offering delivery care, few were close to one that fulfilled BEmOC(−1) or CEmOC(−1) criteria.

Bicycle ownership was fairly common, but motorised transport was virtually absent, with fewer than 1% of births occurring to mothers whose household owned a car or motorbike. These births were much more likely to have been in a facility (19 out of 31 in total). Births in the dry season were somewhat more likely to have taken place in a facility compared to those in the rainy season (Table 2).

**Table 2:** Distribution of physical accessibility determinants and level of delivery care, and crude associations with facility birth in rural Zambia.

Determinants	Births in Category (%) (*n* = 3,692)	Facility Births (%) (*n* = 3,682)	Crude Odds Ratio^a^ (95% CI)
**Season of birth**			*p* = 0.05
Rainy (Nov–May)	56.9	31.5	1
Dry (June–Oct)	43.2	34.0	1.25 (1.00 to 1.56)
**Household transport means**			*p* = 0.02
None	41.7	32.3	1
Bicycle	57.4	32.3	1.24 (0.94 to 1.65)
Motorised	0.8	61.3	5.94 (1.46 to 24.2)
**Distance to closest delivery care** b			*p*<0.001
>15 km	14.1	23.6	1
10–15 km	19.0	24.6	1.35 (0.53 to 3.41)
5–10 km	28.4	30.4	2.04 (0.86 to 4.80)
2–5 km	27.1	42.1	5.76 (2.41 to 13.8)
<2 km	11.4	39.2	4.43 (1.61 to 12.2)
**Level of delivery care within 15 km**			*p*<0.001
None	14.1	23.6	1
Substandard	20.2	29.2	1.85 (0.74 to 4.63)
BEmOC−4	20.8	26.4	1.68 (0.68 to 4.14)
BEmOC−2	16.1	36.5	3.66 (1.39 to 9.62)
BEmOC(−1)	15.9	36.0	3.51 (1.35 to 9.12)
CEmOC(−1)	12.9	48.2	7.63 (2.87 to 20.2)

BEmOC(−1), basic emergency obstetric care that may lack assisted vaginal delivery; BEmOC−2 and −4, facilities lacking two or four of the BEmOC signal functions, respectively; CEmOC(−1), comprehensive emergency obstetric care that may lack assisted vaginal delivery. For details on the classification, see Methods.

a From model adjusting for clustering by sampling cluster and by mother; *p*-values from Wald tests.

b Variable presented in categories for ease of presentation, continuous variable used in regression.

Proximity to delivery facilities was strongly associated with facility birth, as was higher level of obstetric emergency care available within 15 km, both showing a clear trend (Table 2).

In the crude logistic regression model, we found a decrease in the odds of facility delivery by 45% for each unit increase in log-distance (Table 3, Model 1). This corresponds to a 36% decrease in odds of facility delivery for each doubling of distance (0.69 units increase in log-distance).

**Table 3:** Associations of distance and level of delivery care with health facility delivery in rural Zambia.

Models and Variables of Interest	Odds Ratio	95% CI	*p*-Value
**Model 1a: Distance and level of care (categorical)**			
Log-distance to closest delivery care (linear effect)	0.55	0.41 to 0.74	<0.001
Closest facility is substandard level of care (baseline)	1	—	0.001
Closest facility (or within 10 km) is BEmOC−4	1.28	0.62 to 2.63	
Closest facility (or within 10 km) is BEmOC−2	1.77	0.82 to 3.85	
Closest facility (or within 10 km) is BEmOC(−1)	3.23	1.51 to 6.92	
Closest facility (or within 10 km) is CEmOC(−1)	3.99	1.85 to 8.61	
**Model 1b**: **Distance and level of care (linear trend)**			
Log-distance to closest delivery care (linear effect)	0.54	0.40 to 0.73	<0.001
Level of care of closest facility (linear effect over categories, evidence of non-linearity: LRT p = 0.93)	1.45	1.22 to 1.72	<0.001
**Model 2a: Distance and level of care (categorical) adjusted for individual- and household-level confounders** (mother's education, household wealth, ethnic group)			
Log-distance to closest delivery care (linear effect)	0.56	0.43 to 0.74	<0.001
Closest facility is substandard level of care (baseline)	1	—	0.002
Closest facility (or within 10 km) is BEmOC−4	1.19	0.62 to 2.30	
Closest facility (or within 10 km) is BEmOC−2	1.89	0.92 to 3.89	
Closest facility (or within 10 km) is BEmOC(−1)	2.42	1.20 to 4.90	
Closest facility (or within 10 km) is CEmOC(−1)	3.64	1.80 to 7.35	
**Model 2b: Distance and level of care (linear trend) adjusted for individual- and household-level confounders**			
Log-distance to closest delivery care (linear effect)	0.56	0.43 to 0.74	<0.001
Level of care of closest facility (linear effect over categories, evidence of non-linearity: LRT *p* = 0.96)	1.39	1.19 to 1.63	<0.001
**Model 3a: Distance and level of care (categorical) additionally adjusted for cluster-level confounders** (men's fertility attitudes, women's media use, women's relationship autonomy)			
Log-distance to closest delivery care (linear effect)	0.63	0.48 to 0.81	<0.001
Closest facility is substandard level of care (baseline)	1	—	0.06
Closest facility (or within 10 km) is BEmOC−4	0.97	0.52 to 1.82	
Closest facility (or within 10 km) is BEmOC−2	1.32	0.64 to 2.70	
Closest facility (or within 10 km) is BEmOC(−1)	1.49	0.74 to 3.02	
Closest facility (or within 10 km) is CEmOC(−1)	2.51	1.24 to 5.07	
**Model 3b: Distance and level of care (linear trend) additionally adjusted for cluster-level confounders**			
Log-distance to closest delivery care (linear effect)	0.62	0.47 to 0.80	<0.001
Level of care of closest facility (linear effect over categories, evidence of non-linearity: LRT *p* = 0.72)	1.26	1.07 to 1.48	0.005

*n* = 3,682 births; BEmOC(−1), basic emergency obstetric care that may lack assisted vaginal delivery; BEmOC−2 and −4, facilities lacking two or four of the BEmOC signal functions, respectively; CEmOC(−1), comprehensive emergency obstetric care that may lack assisted vaginal delivery. For details on the classification, see Methods.

LRT, likelihood ratio test.

Increasing level of care offered at the closest delivery facility was crudely associated with large increases in the odds of facility delivery. Births whose closest facility offered CEmOC(−1), as opposed to substandard care, had four times the odds of occurring in a facility, adjusting for distance (Table 3, Model 1).

The associations of distance and level of care with facility delivery were attenuated when adjusted for individual- and household-level confounders (Table 3, Model 2), and attenuated further when additionally adjusted for cluster-level confounders (Table 3, Model 3). There was no evidence that the association with distance was modified by level of care (*p* = 0.71), household transport means (*p* = 0.80), or season of birth (*p* = 0.16).

**Figure 3:**
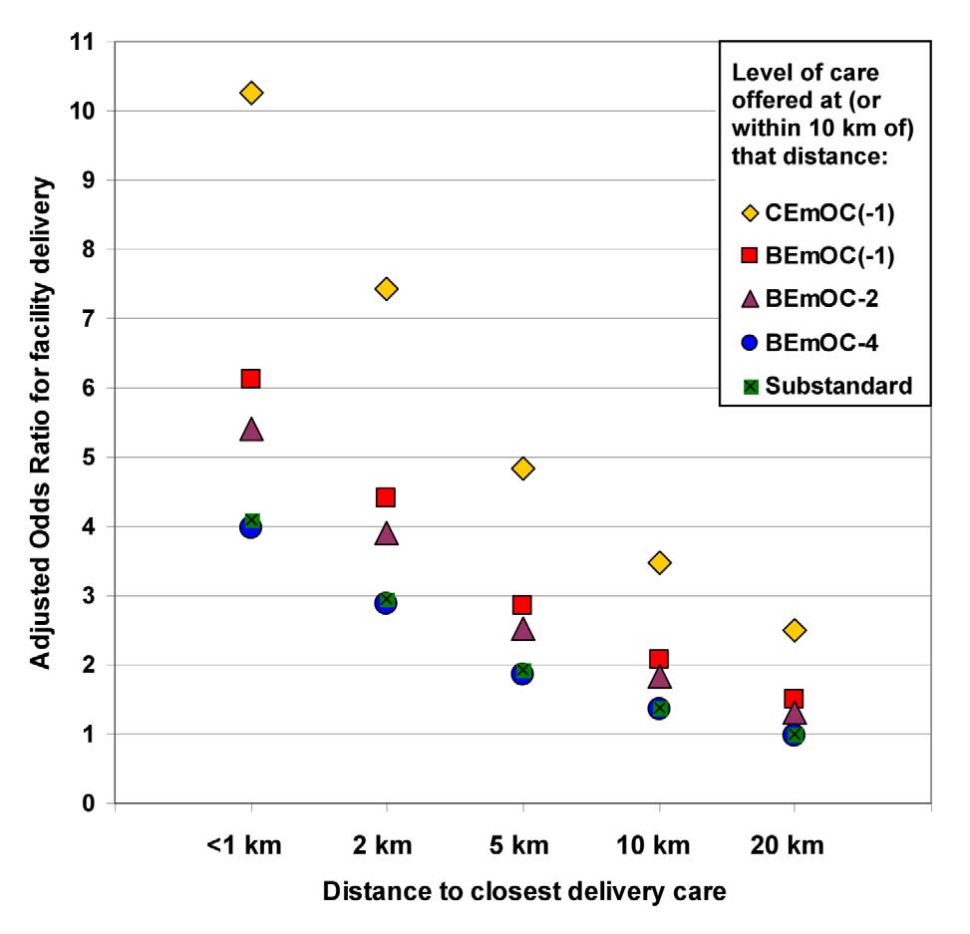
Effects of distance and level of care on health facility delivery in rural Zambia. This graph simultaneously depicts the effects of distance to delivery care and of the level of care offered at or near the closest facility on whether a birth was delivered at a health facility or at home in rural Zambia (Model 3a in Table 3; 3,682 births), adjusted for individual-, household-, and cluster-level confounders (mother's education, household wealth, ethnic group, men's fertility attitudes, women's media use, women's relationship autonomy). The odds of facility birth are higher if the closest facility offers better care: CEmOC (yellow diamonds) higher than BEmOC (red squares), higher than more limited services. For each given level of care, there is a strong effect of distance: The further away, the less likely a birth is delivered in a facility. Births to women living within 1 km of a CEmOC facility have 10 times higher odds of being delivered in a facility than births to women whose closest facility is 20 km away and substandard. BEmOC(−1), basic emergency obstetric care that may lack assisted vaginal delivery; BEmOC−2 and −4, facilities lacking two or four of the BEmOC signal functions, respectively; CEmOC(−1), comprehensive emergency obstetric care that may lack assisted vaginal delivery. For details on the classification, see Methods.

The final, fully adjusted model showed a 29% decrease in odds of facility delivery for every doubling of distance, and a 26% increase in odds of facility delivery for every step increase in level of EmOC, assuming a linear effect (Table 3, Model 3b). Figure 3 depicts Model 3a graphically for distinct distances, showing that the odds of facility delivery for a birth within 1 km of a CEmOC(−1) facility are over 10 times those of a birth whose closest facility is 20 km away and substandard.

Table 4 shows PAFs for distance to a facility offering at least BEmOC−1, as well as for three other determinants of delivery service use: education, wealth, and women's autonomy in the community. PAFs take into account both how common a risk factor is (prevalence) and its relative importance (confounder-adjusted odds ratio), both presented in Table 4, and they thus reflect the absolute importance of these risk factors in rural Zambia.

**Table 4:** Prevalence, effect on home delivery and adjusted PAFs for four determinants of home delivery in rural Zambia.

Variables	Births per category (%)	Adjusted Odds Ratio^a^	PAF in % (95%CI)
**Distance to BEmOC−1**		*p*<0.001	
<5 km	7.4	1	—
5–15 km	21.3	1.50	2.7 (−0.1 to 5.3)
>15 km	71.3	1.90	13.4 (5.7 to 20.4)
**Education**		*p* = 0.001	
Any secondary school	12.9	1	—
Complete primary school	19.0	1.09	0.5 (−1.1 to 2.0)
Incomplete primary school	50.2	1.26	3.3 (−0.6 to 7.1)
No schooling	17.9	1.89	2.9 (1.3 to 4.4)
**Household wealth (asset score)**		*p* = 0.008	
40–88	6.1	1	–
30–39	9.1	1.03	0.1 (−1.2 to 1.4)
20–29	25.1	1.43	2.7 (−0.4 to 5.8)
10–19	37.8	1.63	5.4 (0.9–9.6)
0–9	22.0	1.80	3.6 (0.9 to 6.2)
**Women's relationship autonomy in the community**		*p* = 0.001	
High	12.1	1	—
Medium	39.8	1.50	5.0 (−0.3 to 10.1)
Low	34.6	1.84	6.1 (1.6 to 10.4)
Very low	13.5	2.74	3.5 (1.6 to 5.3)

*n* = 3,594 births, due to missing values in some of the confounders.

a Odds ratio for home delivery adjusted for all other variables that were independent determinants of delivery service use: mother's age at birth, ethnic group, fertility attitudes, family composition, exposure to media health programmes, birth order, previous stillbirth, previous C-section, previous newborn death, twin pregnancy, mother's occupation, husband's occupation, whether getting money is a big problem for care-seeking, men's average fertility attitudes in the cluster, and women's average care-seeking autonomy in the cluster.

**Figure 4:**
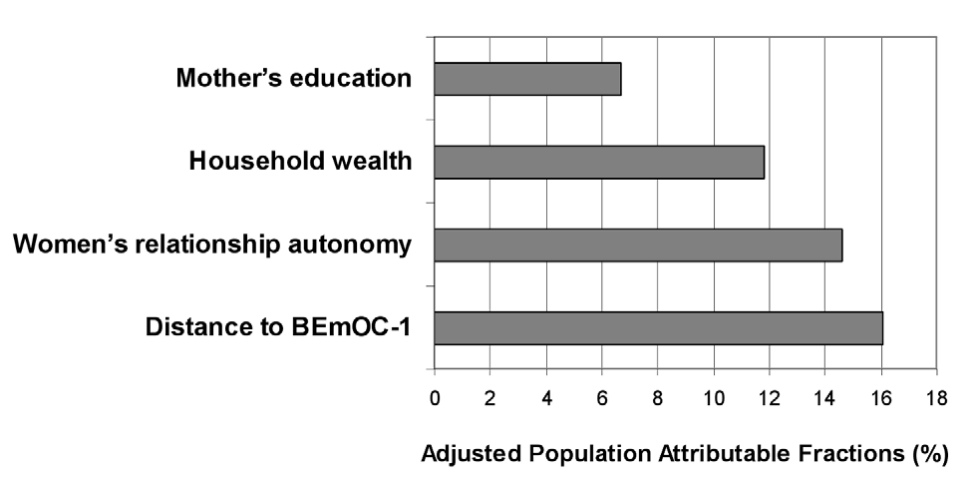
Adjusted PAFs in rural Zambia. PAFs for four determinants of home delivery were computed from an explanatory multivariable logistic regression model including 3,594 births. Assuming causality, this graph depicts the proportion of home deliveries that could be avoided if all births were to women living within 5 km of BEmOC−1, having some secondary education, being least poor, or having highest female relationship autonomy in their community, respectively, adjusting for the other factors and for confounders.

Under the assumption that the associations are causal, these PAFs estimate what proportion of home births could be avoided if women were in the lowest risk groups. If all births were to women living within 5 km of BEmOC−1, 16% of home deliveries could be avoided. This is a comparable order of magnitude as the PAFs for wealth, education, and women's autonomy (Figure 4).

## Discussion

Linking national health facility data with national household survey data in a geographic information system allowed us to redress the lack of adequate data that has so far hampered detailed epidemiological studies on how characteristics of the health services influence use of delivery services [6],[9],[12],[25]. We quantified the strong influence of the health service environment on women's use of health facilities for delivery in rural Zambia, while adjusting for other important determinants on the individual, household, and community level.

We found that for each doubling of distance to the closest delivery facility, the odds of facility birth decreased by 29%, while each step increase in level of obstetric care led to 26% higher odds of facility birth. We also showed that the vast majority of rural Zambian women live far from a facility offering EmOC, and that the population impact of distance to EmOC on place of delivery is as large as that of education or wealth.

The main strengths of this study are the national scope of its detailed health facility information, which permitted a wide range of distances and levels of care to be compared, and its methodological rigour, in particular the consideration of a large range of potential confounders.

So far, very few quantitative studies have assessed the influence of quality of obstetric care on delivery service use, and most did not find evidence of an effect [13],[26]–[29]. In contrast, and in line with our results, many qualitative studies consistently found that quality of care is an important determinant of delivery care-seeking [7]. This discrepancy may be partly due to methodological problems in quantifying quality of care and to a lack of variation within study samples. Quality measures employed so far include reported satisfaction levels (although the problem should be acknowledged that some women may have answered more positively than is true out of courtesy, thereby causing some reporting bias), facility infrastructure, obstetric equipment and drugs, and health-care worker density [13],[26]–[29]. While our measure of level of care captures only some aspects of quality (namely, facility infrastructure, equipment and drugs, and health-care worker availability), no previous study, to our knowledge, evaluated the influence of emergency obstetric care functioning of facilities on use of delivery services.

Most previous studies investigating the influence of distance on delivery service use found it to be an important determinant [7]. However, many of these studies suffered from methodological limitations, such as inadequate control of confounding (in particular not considering confounders at the community level), failure to take clustered data structure into account, and disregard of the fact that some mothers moved residence after the birth. This study, in contrast, adjusted for a wide range of confounders at all levels, excluded movers, and used a three-level random-effects model to account adequately for the dependence of births to the same mother and in the same village. As mentioned already, previous studies often inquired only about distance to the closest facility without considering whether that facility actually provided delivery care, or they collected data in small areas, where geographic access may be relatively homogeneous and findings difficult to generalise.

It is worth noting that odds ratios in our logistic three-level random-effects model should be interpreted as the change in odds of facility delivery for births to a particular mother in a particular village if that mother/village was at a different distance. Such conditional odds ratios are always more extreme than the corresponding marginal odds ratios.

There are several limitations to this study. Distances are likely to contain measurement errors for a number of reasons: some facilities may have been missed by the HFC (especially private facilities; however, these accounted for only 0.1% of births in our rural sample), and for other facilities, geographic coordinates may be incorrect or missing, Macro International adds error to the DHS cluster coordinates to protect participant confidentiality [22], individual households may be far from the cluster centre, and straight-line distance ignores difficulties of terrain. As it seems reasonable to assume that these errors occur independently of the outcome, they will tend to lead to an underestimation of the effect of distance.

Level of obstetric care is likely to also suffer from nondifferential misclassification, as measurement was at only one point in time in 2005 and services may have changed (births occurred between 2002 and 2007), and we are relying on a number of assumptions in terms of actual EmOC provision. While the level of care is an important component of quality of care, to assess quality of care comprehensively, other aspects ideally would be measured as well, including provider competence, adherence to guidelines, and quality of client–provider interaction and communication. Moreover, it would have been desirable to consider not only capability to provide emergency care, but also regular obstetric care, as well as neonatal care. The effect of quality of care is thus likely to be even larger than we have estimated for level of care.

Furthermore, we lacked information on the exact facility women used for delivery, which is recorded in the DHS questionnaires but not entered or released, thus precluding analysis of the possible bypassing by women of lower-level facilities, which may have led to underestimation of the effect of distance. We also lacked information on whether women originally intended to deliver in a facility or sought care only after encountering delivery complications, so we could not separately determine the importance of distance and level of care for preventive and emergency care-seeking. Moreover, we did not have information on cost of care at the facilities. User fees were abolished in rural facilities in Zambia in April 2006 [30]. Most of the births considered in this analysis happened before this date and thus fees charged may have influenced care-seeking. Finally, the DHS does not record information on stillbirths, only on live births, which may have caused selection bias.

Despite its limitations, this study clearly shows that it is important to consider the health service environment when studying use of delivery services, as both distance to services and their quality are important determinants. Ignoring these influential factors can lead to an incomplete picture and invalid conclusions. Their population impact is also substantial, although the absolute PAF estimates should not be overinterpreted, given the data limitations and assumptions of this study. Building, staffing, and ensuring functionality of health facilities, while not easy nor cheap, is attainable and falls within the remit of the health sector. It is certainly also important to address factors such as women's autonomy and education, but without accessible health services that can save lives, other efforts to decrease maternal mortality will be futile. It is therefore crucial that research and policy focus on health system determinants and in particular address geographic and quality barriers to obstetric care.

The increasing availability of georeferenced data provides a promising opportunity to overcome previous data limitations. Our innovative approach of linking large-scale datasets using geographic coordinates could be applied beneficially also in other settings and fields.

Our research suggests that women and their families do make assessments of some aspects of quality and that these assessments influence the distance they are willing to travel. Future studies could investigate how this information on quality of care in facilities is obtained, which aspects in particular influence care-seeking, and how these relate to clinical measures of quality of care. It would also be interesting to investigate whether availability of motorised transport modifies the effect of distance, a potentially important interaction this study lacked power to detect due to the small number of households with motorised transport in rural Zambia.

Ultimately, it would be desirable to go beyond determinants of health facility use and investigate the effect of access to EmOC on maternal mortality, stillbirths, and early neonatal mortality.

## Supporting Information

Figure S1 Proportion of facility births by distance to closest delivery care, untransformed (A) and log-transformed (B). Both plots show average facility delivery by distance to closest delivery care in kilometers, adjusted for confounders (Model 3b from Table 3) using locally weighted regression (lowess smoothing for multiple predictors, user-written command *mlowess* in Stata) for untransformed distance (A), and log-transformed distance (B). The logarithmic transformation renders the association approximately linear. Lowess smoothing does not provide confidence intervals, which would be wide for distances above 20 km, as these are represented by few births (see Figure 2).
(0.18 MB TIF)

## Abbreviations

BEmOC: basic emergency obstetric care
CEmOC: comprehensive emergency obstetric care
CI: confidence interval
DHS: Demographic and Health Survey
EmOC: emergency obstetric care
GIS: geographic information system
HFC: Health Facility Census
PAF: population attributable fraction
PCA: principal component analysis
