# Supplementary figures and images for: The Influence of Distance and Level of Care on Delivery Place in Rural Zambia: A Study of Linked National Data in a Geographic Information System
Source: PLoS Med. 2011 Jan 25;8(1):e1000394. doi: 10.1371/journal.pmed.1000394 (PMC3026699; doi:10.1371/journal.pmed.1000394)

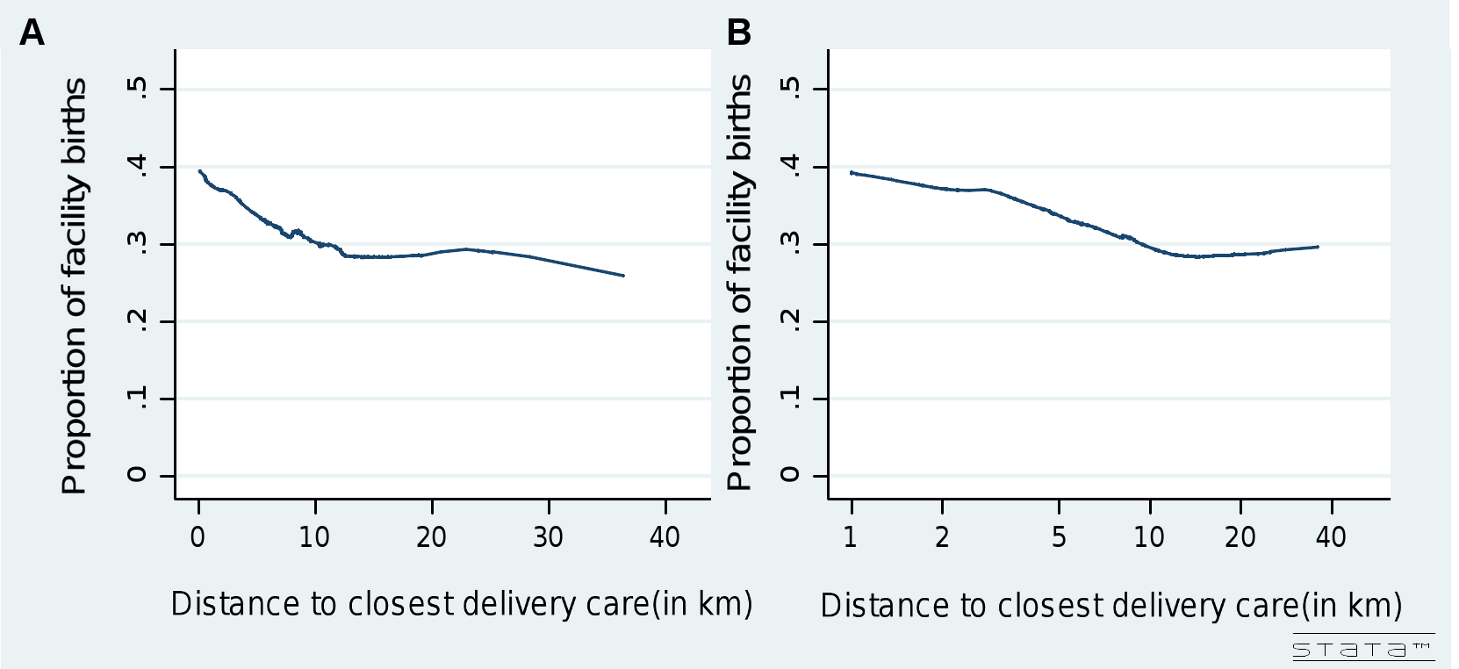

Supplement: Figure S1 — Proportion of facility births by distance to closest delivery care, untransformed (A) and log-transformed (B). Both plots show average facility delivery by distance to closest delivery care in kilometers, adjusted for confounders (Model 3b from Table 3) using locally weighted regression (lowess smoothing for multiple predictors, user-written command mlowess in Stata) for untransformed distance (A), and log-transformed distance (B). The logarithmic transformation renders the association approximately linear. Lowess smoothing does not provide confidence intervals, which would be wide for distances above 20 km, as these are represented by few births (see Figure 2). (0.18 MB TIF) [file pmed.1000394.s001.tif]
